# Long-term follow-up of breast cancer survivors with post-mastectomy pain syndrome

**DOI:** 10.1038/sj.bjc.6602304

**Published:** 2005-01-11

**Authors:** L Macdonald, J Bruce, N W Scott, W C S Smith, W A Chambers

**Affiliations:** 1 Department of Anaesthesia, Aberdeen Royal Infirmary, Foresterhill, Aberdeen AB25 2ZN, UK; 2 Department of Public Health, University of Aberdeen, Polwarth Building, Medical School, Foresterhill, Aberdeen AB25 2ZD, UK

**Keywords:** breast cancer, mastectomy, post mastectomy pain, chronic pain

## Abstract

Post-mastectomy pain syndrome (PMPS) is a recognised complication of breast surgery although little is known about the long-term outcome of this chronic pain condition. In 1996, Smith *et al* identified a prevalence rate of PMPS of 43% among 408 women in the Grampian Region, Northeast Scotland. The aim of this study was to assess long-term outcome at 7–12 years postoperatively in this cohort of women, to describe the natural history of PMPS and impact of pain upon quality of life. Chronic pain and quality of life were assessed using the McGill Pain Questionnaire (MPQ) and Short Form-36 (SF-36). Of 175 women reporting PMPS in 1996, 138 were eligible for questionnaire follow-up in 2002. Mean time since surgery was 9 years (s.d. 1.8 years). A response rate of 82% (113 out of 138) was achieved; 59 out of 113 (52%) women reported continued PMPS and 54 out of 113 (48%) women reported their PMPS had resolved since the previous survey in 1996. Quality of life scores were significantly lower in women with persistent PMPS compared to those women whose pain had resolved. However, for women with persistent PMPS, SF-36 scores had improved over time. Risk factors for persistent PMPS included younger age and heavier weight. This study found that, of women reporting PMPS in 1996, half of those surveyed in 2002 continued to experience PMPS at a mean of 9 years after surgery.

Post-mastectomy pain syndrome (PMPS) is a chronic pain condition, typically neuropathic in nature, which can occur following surgery to the breast. Persistent pain after mastectomy was first reported during the 1970s, characterised as a dull, burning and aching sensation in the anterior chest, arm and axilla, exacerbated by movement of the shoulder girdle (Wood, 1978). More recently, chronic pain has been reported after other breast procedures, including lumpectomy, breast reconstruction, augmentation and reduction (Wallace *et al*, 1996; Carpenter *et al*, 1998). The exact cause of PMPS is unclear, but various aetiological theories have been postulated, including dissection of the intercostobrachial nerve, intraoperative damage to axillary nerve pathways and pain caused by neuroma (Vecht *et al*, 1989; Wallace *et al*, 1996; Jung *et al*, 2003).

There is no standard definition for PMPS, although the International Association for the Study of Pain has defined chronic pain as that persisting beyond the normal healing time of 3 months (IASP, 1986). The frequency of PMPS ranges from 4 to 56%, depending on the definition used and method of assessment (Vecht *et al*, 1989; Tasmuth *et al*, 1995; Wallace *et al*, 1996). Although evidence regarding the epidemiology of PMPS is growing, little is known about risk factors for the development of PMPS, or the long-term outcome of this chronic pain condition (Jung *et al*, 2003).

In 1996, an epidemiological study was conducted to determine the prevalence of chronic pain in women undergoing mastectomy for breast cancer in the Grampian Region, Northeast Scotland (Smith *et al*, 1999). A cumulative prevalence of 43% (175 out of 408 women) was reported at three years (mean) postoperatively. The aim of this follow-up study was to investigate the long-term outcome in the 175 women who had reported PMPS. Specific objectives were to assess the long-term prognosis, risk factors and quality of life in this cohort of surviving women who were between 7 and 12 years after surgery at the time of second follow-up.

## MATERIALS AND METHODS

### Study design and sample size

A cohort study design was used to assess the outcome of women who underwent a mastectomy procedure (OPCS-4 Operation Codes B272–B279) in Grampian University Hospitals Trust between 1990 and 1995. Six categories of mastectomy were included: total, subcutaneous, excision of both pectoral muscles, excision of pectoral minor, other and unspecified. A total of 408 women were surveyed in 1996; of these, 175 women reported PMPS and were eligible for follow-up in 2002. Of the 175 women reporting PMPS, 149 (85%) had total mastectomy. Ethical approval for the follow-up study was granted from the Grampian Research Ethics Committee of NHS Grampian and University of Aberdeen.

### Tracing process and study procedures

The Community Health Index unique identifier was used by the Grampian Health Board to check vital status, current address and general practitioner (GP) for the 175 women. Patients now residing outside of the Grampian Region were not traced. Letters were sent to GPs for each patient, to inform them of the study and to avoid inappropriate enquiry.

### Definition and measurement of chronic pain

Chronic pain was defined as PMPS based on three criteria: character, location and timing of pain. The same criteria for PMPS were used at both time points to ensure comparability. Pain nature was assessed for neuropathic characteristics, including numbness, pins and needles, burning or stabbing. Pain location was recorded as the same side of surgery, chest wall, axilla or ipsilateral arm. Timing of pain was defined as persisting either continuously or intermittently for longer than the normal healing time of 3 months (IASP, 1986). Cumulative prevalence accounts for variability of pain onset and duration, thus indicating the number of women with persistent or intermittent PMPS between the two survey time points. Point prevalence was used to denote women reporting PMPS on the actual day of second survey.

### Questionnaire design

The survey instrument was based on the baseline PMPS questionnaire with minor modifications to account for changes in timing (Smith *et al*, 1999). Quality of life was assessed using the Short Form-36 (SF-36) (Ware *et al*, 1993). Pain characteristics were quantified using the McGill Pain Questionnaire (MPQ) and the University of California and San Francisco (UCSF) Pain Service questionnaire (Melzack, 1983; Fields, 1987). Patients were asked to complete upper body pain charts using different graphical symbols for numbness, pins and needles, burning, stabbing and ache (Boureau *et al*, 1990). Free-text sections were included for women to describe their experience with pain, limitations of daily activities and coping strategies. Questionnaires were sent with a covering letter and stamped addressed envelope; one reminder was sent after 3 weeks.

### Statistical analysis

Data were analysed using SPSS for Windows (version 11.0). *P*-values of less than 0.05 were considered statistically significant. The MPQ was analysed using three methods as described by McDowell and Newell (1987): the Pain Rating Index using scale weight values (PRI); the Pain Rating Index using scale weighted-rank values (PRI-R); the number of words chosen (NWC) as per analysis of the baseline data (Bruce *et al*, 2004). For the UCSF questionnaire, numbers of women with symptoms were reported. For ordered categorical variables, the *χ*^2^ test for trend was used. Independent-samples *t*-tests were used for comparing pain and pain-free groups. Paired *t*-tests were used to compare the mean MPQ and SF-36 values over time. Multiple regression was used to compare SF-36 domains across pain groups; analysis was adjusted for age to account for known relationship between increasing age and lower quality of life. Standard syntax was used to calculate SF-36 scores where responses were summed and transformed to generate dimension scores ranging from 0 (poor health) to 100 (excellent health).

## RESULTS

### Sample tracing and response rate

**Figure 1:**
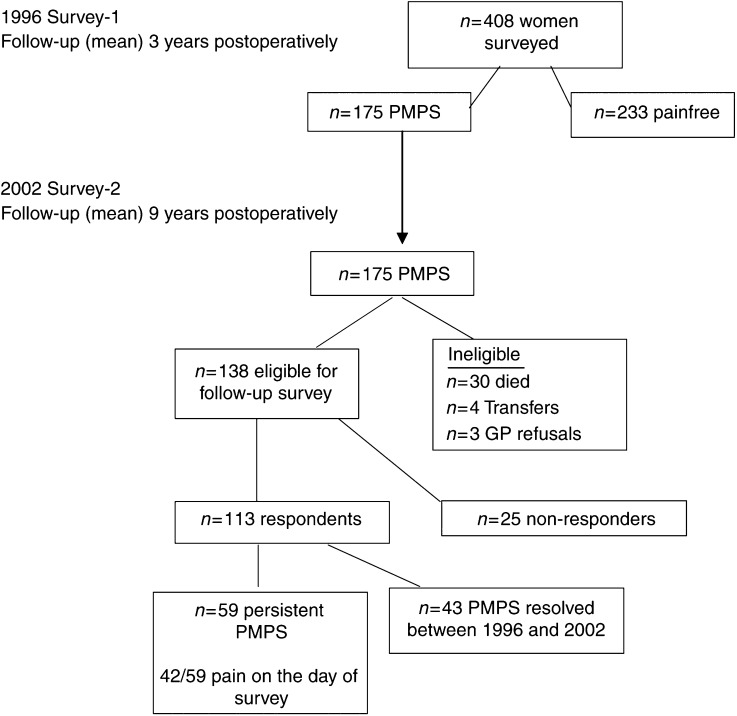
Flowchart of surveys and follow-up rates.

Of the 175 women with PMPS eligible for follow-up in 2002, 30 patients had died, four had transferred out of Grampian Region and three GPs refused further contact. A questionnaire survey was administered to 138 eligible women; 121 (88%) were returned, of these eight were blank or incomplete. The results are based on 113 out of 138 responses with complete data (82%) (Figure 1).

### Characteristics of responders

The mean time since breast surgery was 9.1 years (s.d. 1.8 years). The mean age of women at follow-up was 62 years (s.d. 10.5 years). Most women were currently married (64%) and a quarter of responding women (25%) lived alone. Of the 113 women reporting persistent PMPS, 96 (85%) had undergone a total mastectomy; 13 (12%) total mastectomy with excision of both pectoral muscles; four (3%) had undergone subcutaneous mastectomy.

### Prevalence of persistent PMPS

Of 113 women who reported PMPS in 1996, 59 (52%) women reported having PMPS since that time, 43 (38%) reported their pain had resolved and 11 (10%) had pain elsewhere unrelated to surgery (e.g. arthritis, back pain). The cumulative prevalence of PMPS at a mean of 9 years postoperatively was 52% (59 out of 113) for the follow-up sample. A total of 42 of 113 women reported having PMPS on the day of the 2002 survey, a point prevalence rate of 37%. Of the 59 women reporting persistent PMPS, 45 reported problems when using their arm and 24 still had arm swelling on the side of breast surgery.

The previous survey in 1996 had estimated that 175 out of 408 (43%) of women had PMPS at a mean of 3 years after surgery. Excluding 62 patients lost to follow-up (dead, not traced, refusals and nonrespondents) from the 408 denominator and assuming that the 233 women who were painfree in 1996 did not subsequently develop PMPS, our second survey suggests that 59 out of 346 (17%) women will have PMPS at some point between a mean of 3 and 9 years after surgery (range 7–12 years).

### Risk factors for persistent PMPS

Women with persistent PMPS up to 2002 were younger than those whose pain had resolved (mean age 49.5 *vs* 56.2 years; *t*-test; *P*<0.001). The frequency of PMPS decreased with age from 91% in women aged 30–49 years, to 55% in women aged 50–69 years, to 29% in women aged 70 years and over (*χ*^2^ test for trend; *P*<0.001). Women with persistent PMPS were significantly heavier than women whose pain had resolved by 2002 (Table 1). No significant differences were found in body mass index between those with persistent and resolved chronic pain, or in the mean time since breast surgery between the two groups (Table 1).

### Characteristics of persistent PMPS

The MPQ pain scores reported in 1996 and 2002 were compared; a total of 47 out of 59 women reporting PMPS (80%) completed the MPQ at both time points. A nonsignificant reduction in PRI and PRI-R scores was noted over time, suggesting a decrease in pain intensity (Table 2). The mean number of adjectives chosen to describe PMPS was significantly lower in 2002 compared to 1996 (7.4 *vs* 5.9; *P*=0.04, Table 2). Sensory-discriminative descriptors were chosen more frequently than motivational-affective, cognitive-evaluative or miscellaneous terms. The most frequently selected MPQ terms were: stabbing (*n*=20), shooting (*n*=17), aching (*n*=17), gnawing (*n*=14), nagging (*n*=14), annoying (*n*=13), numb (*n*=13), tiring (*n*=13) and tight (*n*=12). All of the 17 women who reported ache also selected other pain descriptors.

Analysis of the UCSF questionnaire was undertaken: 21 out of 59 (36%) of women reporting pain in 2002 described the intensity of their pain as variable, 12 reported a decrease in intensity and four reported that pain had increased since it first started (*n*=22 missing). Sleep was unaffected for most women (*n*=30), but others reported sleep was interrupted one to two nights per week (*n*=11) or for more than three nights per week (*n*=10). Pain was predominantly aggravated by straining (*n*=42), lying down (*n*=11), cold weather (*n*=8), clothes rubbing against the scar (*n*=8) and getting out of bed (*n*=8). The pain was improved by sitting (*n*=9) and, for some women, lying down (*n*=11). Activities such as lifting, carrying and general housework caused problems. Stretching movements were described as exacerbating the pain, and exercise was difficult, although some women used exercise and physiotherapy in their treatment regimes. Women described problems in daily activities that are usually ‘taken for granted’, like walking, driving and working.

### Treatment of PMPS

Of the 59 women with persistent PMPS, most had tried one or more treatments for pain relief (*n*=53). The most commonly tried method was conventional analgesia, either from a pharmacy or prescribed by a GP (*n*=35). Other women had tried other nonpharmaceutical treatments or a combination of therapies, such as physiotherapy, homeopathy, reflexology, reiki, acupuncture, massage and counselling. Women described these therapies as being helpful for ‘dealing with’ the pain; some also described creating their own strategies for how best to live with chronic pain. Free-text descriptions of alternative approaches included ‘mind over matter’, ‘sunny holidays’ and ‘just getting away from it all’.

### Quality of life

Women with persistent PMPS up to 2002 had statistically significantly lower SF-36 scores across all health domains, except for role-emotional functioning, than women whose pain had resolved since assessment in 1996. These findings persisted when these results were adjusted for age using multiple regression (Table 3). For those women reporting persistent PMPS, self-reported quality of life was compared between the two survey time points, 1996 and 2002 (mean 3–9 years postoperatively). Mean scores had improved in all but one domain, role-emotional functioning, and there was a statistically significant improvement in physical functioning (Table 4).

## DISCUSSION

This study reports on the long-term prognosis and quality of life in a cohort of women who reported chronic pain after breast surgery. A cumulative prevalence of 43% (175 out of 408) was identified in 1996 at a mean of 3 years after mastectomy surgery; 59 of the 113 (52%) women who completed questionnaires at follow-up in 2002 reported having continuing PMPS. Using the denominator of 346 women (408 original sample minus 62 losses to follow-up), our study suggests that 17% (59 out of 346) women will have PMPS up to 12 years postoperatively (mean 9 years). We cannot exclude the possibility that women who were painfree in 1996 may have since developed PMPS, although this is unlikely. Cumulative prevalence was used to allow for the variability of onset and remission of pain. However, a high-point prevalence rate was found in women who were followed up, with 42 of 113 (37%) women reporting PMPS on the day of survey in 2002, which is considerable given they had breast surgery on average 9 years previously.

There is current debate over the definition and diagnosis of neuropathic pain, currently defined by IASP as ‘pain initiated or caused by a primary lesion or dysfunction in the nervous system’ (Merskey and Bogduk, 1994; Max, 2002). This definition focuses on aetiology, thus is not applicable when categorising pain status based on self-reported symptoms using questionnaire methodology. We used a rigorous definition for PMPS based on nature (neuropathic descriptors), location and timing of pain, as per the 1996 survey (Smith *et al*, 1999). In a comprehensive review of neuropathic pain after breast cancer surgery, Jung *et al* (2003) proposed four distinct pain syndromes: phantom breast pain, intercostobrachial neuralgia (ICN), neuroma pain and other nerve injury pain. Intercostobrachial neuralgia pain is that typically accompanied by sensory changes in the distribution of the intercostobrachial nerve, whereas neuroma pain is found in the region of the scar on the breast, chest or arm, provoked by percussion. It would be unreliable to attempt to categorise the women in our study to these syndromes without detailed neurological assessment. Nevertheless, it is likely that women in our sample have ICN, neuroma pain or a combination of both.

Verbal descriptors of pain symptoms, along with neurological examination, are widely used to diagnose neuropathic pain. Neuropathic pain, therefore, is a categorical description rather than a single entity (Krause and Backonja, 2003). Pain descriptors reported by women in our study were similar to those reported at 3 years postoperatively: stabbing, shooting, aching and nagging (Bruce *et al*, 2004). Although our sample of women completing the MPQ at both time points is small, terms used by women to describe their PMPS are similar to adjectives reported in other studies (Stevens *et al*, 1995). Ache was selected within the MPQ by 30% of women with PMPS in the 2002 survey, although none of the women described their pain as ‘ache’ alone. In 1990, Boureau and colleagues used the French version of the MPQ to differentiate neuropathic pain descriptors from non-neuropathic pain descriptors. The descriptor ‘ache’ was classified as non-neuropathic; this categorisation was adopted in the 1996 PMPS study, whereby women reporting only ache (*n*=39) were excluded from further follow-up. Given the considerable variation in signs and symptoms of neuropathic pain, some of these women may indeed have had nerve-related injury after breast surgery.

Although pain descriptors are used to differentiate between neuropathic and non-neuropathic pain, particularly in population-based studies, few studies have explored the discriminative and predictive ability of specific descriptors. Krause and Backonja recently published a 12-item Neuropathic Pain Questionnaire (NPQ), based on an original 32-item list that included ‘dull, aching pain’. This list was administered to patients with neuropathic and non-neuropathic pain; dull ache was dropped from the final 12-item NPQ; the three-term short form version included ‘numbness, tingling and increased pain due to touch’ (Backonja and Krause, 2003; Krause and Backonja, 2003). In contrast, Galer and Jensen (1997) include ‘dull and aching pain’ within the Neuropathy Pain Scale (NPS).

This is the first study to assess long-term prognosis of PMPS and to explore change in health-related quality of life over time. The SF-36 is a valid measure of change in population health and is also responsive to quality of life changes in patients with chronic pain conditions, for example, low back pain, widespread pain and post-operative pain (Ware, 2000). In general, female sex and increasing age are associated with poorer health-related quality of life, with physical health scores tending to decline greater than mental health (Lyons *et al*, 1994; Walters *et al*, 2001). UK population ‘norms’ for mean SF-36 scores have been published, based on large-scale surveys in English populations (Jenkinson *et al*, 1993; Bowling *et al*, 1999). These surveys found that women aged 60–64 years have higher than expected SF-36 scores, with scores being higher than women in younger and older age categories (Bowling *et al*, 1999; Walters *et al*, 2001). The mean age of women for the Grampian cohort at second survey was 62 years. We found that, overall, quality of life scores improved over time since surgery, particularly for physical functioning. The mean SF-36 scores for our female sample were lower for all health domains compared to standard population scores, although statistical testing was not undertaken.

Younger age and higher BMI were identified as risk factors for persistent pain at a mean of 3 years postoperatively. Younger women were more likely to have persistent pain at 9 years, and although the association with heavier weight remained, BMI was no longer a significant risk factor. The relationship with age is unclear, as younger age has been identified as an independent risk factor in some studies (Tasmuth *et al*, 1995) but not others (Carpenter *et al*, 1998; Jung *et al*, 2003). It could be argued that women with chronic pain may be less physically active, which could contribute to heavier weight, yet mean physical functioning scores improved over time in our sample. Younger age and obesity have been reported as independent risk factors for chronic pain after other surgeries, including inguinal herniorrhaphy and cardiac surgery (Poobalan *et al*, 2001; Bruce *et al*, 2003). Given that the majority of women in our sample underwent total mastectomy, surgical procedure was not assessed as a risk factor. However, we acknowledge that breast surgery has evolved since the 1990s, with breast–conserving procedures, for example, lumpectomy with or without axillary node dissection, now being conducted in up to 40% of breast cancer surgeries (Jung *et al*, 2003).

Although our sample size is small, our data suggest that the intensity of PMPS decreases over time, but perhaps women have also developed adaptation mechanisms or strategies to learn to cope with persistent pain. The free-text data suggested that women were very positive, suggesting they were highly motivated and having survived breast cancer, happy to be alive. Despite having problems with daily activities and having tried various modes of therapies, women described ‘getting on with their lives’, trying to live each day to the full within their own very specific limitations. Our sample had tried conventional analgesia and other treatment approaches, including relaxation, reflexology, reiki and massage, mostly described as helpful in coping with persistent pain. Women also expressed willingness to participate in the study; indeed, a questionnaire response rate of 82% at 9 years postoperatively is remarkable.

Our study is limited by the small sample size, the absence of data on preoperative quality of life, pain intensity and analgesic consumption in the acute postoperative period. Increased pain intensity in the immediate postoperative period after breast surgery is associated with the development of chronic pain (Tasmuth *et al*, 1995). Identification of possible risk factors is an important step in understanding the pathway and processes for the development of chronic post-surgical pain. Future intervention studies should be designed to target specific risk groups and identify those most in need of preventive strategies.

This study reports on the long-term outcome of 175 women assessed at a mean of 3 and 9 years after breast cancer surgery. There was a suggestion of a decrease in the intensity of pain over time although neuropathic characteristics of PMPS remained constant. For women with persistent PMPS up to 2002, there was gradual improvement in quality of life from 3 years postoperatively, although health-related quality of life was poorer compared to women whose PMPS had subsequently resolved. Younger age and heavier weight were independent risk factors for PMPS at both time points. Surgeons should be aware that younger and heavier women may be at a higher risk of developing chronic pain syndromes after breast cancer surgery. All women undergoing breast cancer surgery should be fully informed of the possibility of developing chronic neuropathic pain syndromes.

## Figures and Tables

**Table 1:** Characteristics of women who were surveyed in 2002, 7–12 years after breast surgery (mean 9 years)

	**Persistent PMPS since 1996**		**PMPS resolved between 1996 and 2002**		
	**No. of patients**	**Mean (s.d.)**	**No. of patients**	**Mean (s.d.)**	***P*-value**
Age (years)	59	49.5 (9.8)	54	56.2 (10.9)	0.001
Weight (kg)	46	70.5 (12.4)	42	63.9 (9.1)	0.004
Height (m)	56	1.63 (0.1)	50	1.61 (0.1)	0.17
Body Mass Index	44	26.7 (4.7)	38	24.9 (4.5)	0.09
Time since breast surgery (years)	59	8.9 (1.9)	54	9.1 (1.8)	0.66

**Table 2:** Mean (s.d.) McGill Pain Questionnaire (MPQ) scores for women with persistent PMPS

	**Survey-1 1996**	**Survey-2 2002**		
**MPQ domain**	***N*=47; mean (s.d.)**	***N*=47; mean (s.d.)**	**Mean paired difference^a^ (95% CI)**	***P*-value**
Pain Rating Index (PRI)	18.34 (11.8)	15.76 (12.5)	2.58 (−0.8, 5.9)	0.13
Pain Rating Index-Ranked (PRI-R)	16.17 (10.6)	14.68 (12.4)	1.49 (−1.6, 4.6)	0.33
Number of words chosen (NWC)	7.34 (4.4)	5.96 (4.4)	1.38 (0.1, 2.7)	0.036

a Difference in mean pain scores compared between 1996 and 2002, *t*-test.

**Table 3:** Comparison of mean (s.d.) SF-36 scores in women with persistent and resolved PMPS (assessment 7–12 years after surgery)

**SF-36 domain**	**Persistent PMPS since 1996 (*N*=59)**	**PMPS resolved between 1996 and 2002 (*N*=54)**	**Mean difference (95% CI)^a^**	***P*-value**
Physical functioning	60.4 (30.7)	74.6 (25.0)	−14.2 (−25.1, −3.3)	0.01
Role – physical	54.4 (44.1)	72.4 (40.6)	−18.1 (−34.5, −1.6)	0.03
Bodily pain	55.8 (26.5)	76.9 (21.6)	−21.1 (−30.3, −11.9)	<0.001
General health perceptions	60.7 (23.7)	73.1 (18.4)	−12.4 (−20.7, −4.0)	0.004
Vitality	49.4 (24.4)	64.5 (19.2)	−15.2 (−23.3, −6.9)	<0.001
Social functioning	73.3 (31.2)	86.6 (21.3)	−13.3 (−23, −3.4)	0.009
Role – emotional	69.0 (42.2)	81.3 (37.0)	−12.3 (−27.5, 2.6)	0.11
Mental health	71.4 (21.6)	80.5 (13.7)	−9.1 (−15.9, −2.3)	0.009

a Multiple regression adjusted for age.

**Table 4:** Comparison of mean (s.d.) SF-36 scores over time in women with persistent PMPS between 1996 and 2002

**SF-36 domain**	**No. of women**	**1996 Mean (s.d.) score**	**2002 Mean (s.d.) score**	**Difference of means (95% CI)^a^**	***P*-value**
Physical functioning	57	59.7 (30.5)	67.1 (26.8)	−7.4 (−12.5, 2.2)	0.006
Role – physical functioning	54	54.2 (44.2)	56.5 (42.4)	−2.3 (−13.6, 8.9)	0.68
Bodily pain	58	55.8 (26.5)	61.9 (24.4)	−6.2 (−12.7, 0.4)	0.06
General health perceptions	56	61.6 (23.7)	66.5 (23.7)	−4.94 (−10.3, 0.4)	0.07
Vitality	59	49.4 (24.4)	51.4 (23.2)	−2.09 (−8.7, 4.5)	0.53
Social functioning	59	73.3 (31.2)	79.2 (23.7)	−5.93 (−13.2, 1.3)	0.11
Role – emotional	54	72.8 (39.9)	72.2 (40.3)	−0.62 (−11.8, 13.1)	0.92
Mental health	59	71.4 (21.7)	72.0 (17.3)	−0.61 (−6.5, 5.3)	0.84

a Multiple regression, adjusted for age.
